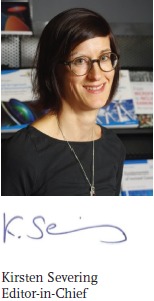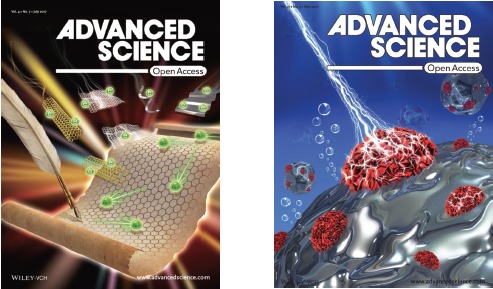# Starting the 5th Volume

**DOI:** 10.1002/advs.201700872

**Published:** 2018-01-17

**Authors:** Kirsten Severing

**Figure 1:**
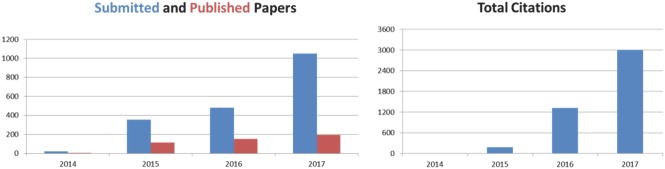
Key performance indicators for Advanced Science during its first years.

Who would have believed it? When we launched *Advanced Science* in December 2014, everyone in the editorial team agreed that it was a fantastic idea to create a forum for top science, open to read, download, and share with everyone, but the development that this young journal has undergone during the last three years has stunned even the most optimistic among us. **Figure** **1** shows some key performance indicators that reflect this great development. The number of submissions has increased by 100% as compared to 2016 and the number of citations has doubled. The impact factor increased by more than 50% to 9.034 in 2017 (Clarivate Analytics, Journal Citation Reports), and for 2018 we are confident of achieving a two‐digit impact factor! Additionally, the number of full text downloads has grown by 50% **Table** **1** shows the highest cited papers in 2017 (published in 2015 and 2016), which will be the driving force for the next impact factor. Here, we find outstanding papers from China and Singapore, but also from Israel, the USA, Australia, and the UK.

**Table 1:** Most cited papers in 2017.

Article title	Corresponding author	Publication date	Total citations	Citations in 2017
A Review of Solid Electrolyte Interphases on Lithium Metal Anode	Qiang Zhang, *Tsinghua University, Beijing, China*	March 2016	133	104
Control of Emission Color of High Quantum Yield CH_3_ NH_3_ PbBr_3_ Perovskite Quantum Dots by Precipitation Temperature	Andrey L. Rogach, *City University Hong Kong, China*	September 2015	126	68
Transition Metal Carbides and Nitrides in Energy Storage and Conversion	Hong Jin Fan, *Nanyang Technology University, Singapore*	May 2016	118	98
Nanoparticle‐Hydrogel Composites: Concept, Design, and Applications of These Promising, Multi‐Functional Materials	Xian Jun Loh, *National University Singapore, Singapore*	February 2015	92	57
Janus Separator of Polypropylene‐Supported Cellular Graphene Framework for Sulfur Cathodes with High Utilization in Lithium‐Sulfur Batteries	Qiang Zhang, *Tsinghua University, Beijing, China*	January 2016	84	55
Advances in Perovskite Solar Cells	David Cahen and Liming Ding, *Weizmann Institute of Science, Israel and National Center of Nanoscience & Technology, Beijing, China*	July 2016	81	62
Recent Progress in Electronic Skin	Caofeng Pan, *Chinese Academy of Science, Beijing, China*	October 2015	75	51
Porous Nickel‐Iron Oxide as a Highly Efficient Electrocatalyst for Oxygen Evolution Reaction	Rui Cao, *Shaanxi Normal University, Xian, China*	October 2015	73	51
Growth of Ultrathin ZnCo_2_O_4_ Nanosheets on Reduced Graphene Oxide with Enhanced Lithium Storage Properties	David Lou, *Nanyang Technology University, Singapore*	February 2015	54	28
Anchoring CoO Domains on CoSe_2_ Nanobelts as Bifunctional Electrocatalysts for Overall Water Splitting in Neutral Media	Bin Zhang, *Tianjin University, Tianjin China*	June 2016	42	39
Biodegradable and Renal Clearable Inorganic Nanoparticles	Weibo Cai, *University of Wisconsin, Madison, USA*	February 2016	39	33
Beyond Traditional RAFT: Alternative Activation of Thiocarbonylthio Compounds for Controlled Polymerization	Greg G. Qiao, *University of Melbourne, Australia*	September 2016	38	32
The Role of Local Triplet Excited States and D‐A Relative Orientation in Thermally Activated Delayed Fluorescence: Photophysics and Devices	Andrew P. Monkman, *University of Durham, England*	December 2016	37	35

The rapid growth in submissions and the high general interest in the journal have resulted in the appointment of two new Deputy Editors. We welcome Anne Pfisterer and Prisca Henheik to the editorial team. Prisca studied chemistry, then earned her Ph.D. in life sciences, and has been with Wiley for 15 years. She is the Editor‐in‐Chief of *Clean* and is an editor of *Advanced Materials*, *Advanced Sustainable Systems*, and *Global Challenges*. Anne is a Deputy Editor for the *Macromolecular* journals and has been responsible for the recent launch of *Advanced Therapeutics*. She has a strong background in polymer science and drug delivery. Coming from the group of Klaus Müllen, her expertise includes energy related topics and for several years she has been an editor of *Advanced Energy Materials*. We are also looking forward to the support of Duoduo Liang, Deputy Editor of *Advanced Materials*. Of course, the rest of the current team is not leaving *Advanced Science*! Without the help of our enthusiastic global editorial team, the success of *Advanced Science* would not have been possible. We thank Esther Levy, Hakim Meskine, Andrew Moore, Lorna Stimson, and Peter Gregory for their fundamental help and support. Special thanks go to Guangchen Xu, who has done a tremendous job promoting the journal in Asia. All of them will stay active in *Advanced Science* as Consulting Editors and will still be open to your feedback and ideas.

Ulf Scheffler, who has been the technical editor for *Advanced Science* for the past two years, is transitioning to the peer review team of our department. I'd like to thank him for running the post‐acceptance work of the journal in his typically efficient and friendly way.

With this, I would like to thank all of you, editorial board members, authors, reviewers, and readers, for your interest in *Advanced Science*. I hope you will follow us on our journey into the exciting fifth volume.

On behalf of the editorial team,